# Histopathological expression analysis of intercellular adhesion molecule 1 (ICAM-1) along development and progression of human melanoma

**DOI:** 10.18632/oncotarget.20884

**Published:** 2017-09-14

**Authors:** Gilli Galore-Haskel, Erez N. Baruch, Amy L. Berg, Iris Barshack, Itzhak Zilinsky, Camila Avivi, Michal J. Besser, Jacob Schachter, Gal Markel

**Affiliations:** ^1^ Ella Lemelbaum Institute of Immuno-Oncology, Ramat-Gan, Israel; ^2^ Clinical Microbiology and Immunology, Tel-Aviv, Israel; ^3^ Institute of Pathology, Ramat-Gan, Israel; ^4^ School of Medicine, Sackler Faculty of Medicine, Tel Aviv University, Tel-Aviv, Israel; ^5^ Department of Plastic and Reconstructive Surgery, Ramat-Gan, Israel; ^6^ Talpiot Medical Leadership Program, Sheba Medical Center, Ramat-Gan, Israel

**Keywords:** melanoma, ICAM-1, adhesion, progression

## Abstract

Intercellular adhesion molecule 1 (ICAM-1) protein is an important adhesion molecule that facilitates metastasis on one hand, and on the other hand supports the immunological synapse necessary for T-cell mediated elimination. The expression pattern of ICAM-1 in melanoma was studied more than two decades ago, mainly in cell lines or in unmatched melanoma specimens. By using real time PCR we could not demonstrate a clear difference in ICAM-1 mRNA levels between primary melanocytes and primary cultures of metastatic melanoma. However, immunohistochemistry staining of progression tissue microarray comprised of samples of different disease stages derived from different patients, demonstrated a dramatic ICAM-1 upregulation particularly upon the transition from primary tumor to lymph node metastasis. There was no significant difference between lymph node and distant metastases. Importantly, these results were confirmed in an independent tissue microarray comprised of patient-paired specimens from progressive stages of the patient’s disease. These data indicate that ICAM-1 upregulation is required to initiate the lymphatic spread of melanoma (Stage III) but no further increase is associated with progression to remote organs (Stage IV).

## INTRODUCTION

Malignant melanoma, arising from pigment producing melanocytes, is the most lethal form of skin cancer. The incidence of melanoma in Caucasian populations has been increasing at a higher rate than any other malignancy [1]. Numerous molecular events have been associated with the development and progression of melanoma by affecting different pathways for proliferation, apoptosis and migration. (reviewed in [2]).

Cell adhesion molecules (CAMs) are cell surface molecules which assist cells to adhere to other cells or to the extracellular matrix. They also allow the exchange of information between cells. Alterations in the function and expression of CAMs results in disruption of normal cell-cell interactions and may lead to malignant transformation and tumor progression (reviewed in [3]). Human melanoma cells have been found to express a number of cell adhesion molecules, among them Intercellular adhesion molecule 1 (ICAM-1), which mediates their interaction with leukocytes [4]. Binding of ICAM-1 to integrin lymphocyte function-associated antigen-1 (LFA-1) is essential for optimal interaction between CTLs and target cells and facilitates T-cell activation [5]. Pandolfi et al. showed that anti-ICAM-1 inhibited the cytotoxicity of tumor infiltrating lymphocytes against autologous melanoma cells [6]. ICAM-1 can be induced by inflammatory cytokines such as IFN-γ, IL-1 and TNF-α [7]. We have previously shown that melanoma cells downregulate the expression of adenosine deaminase acting on RNA (ADAR1) enzyme upon the transition from primary to metastatic melanoma [8]. This renders the melanoma cells more resistant to T-cells by lowering ICAM-1 protein expression [9]. On the other hand, several studies suggested the involvement of ICAM-1 in melanoma progression and prognosis. The expression of ICAM-1 by cells of melanocytic origin increases with the progression of the malignant transformation process [10–13], and a significant association was observed between ICAM-1 expression in primary lesions and the thickness of the lesion as well as with a reduction in disease free survival [11, 14]. In line with these studies, *in vivo* studies showed that suppression of ICAM-1 expression inhibits the metastatic capacity of melanoma cells [15]. Importantly, the expression of ICAM-1 in melanocytic lesions was last studied in 1997, using primarily cell lines which may be significantly biased, and may account for the conflicting data described above. In this work, we analyze the expression of ICAM-1 during melanoma development and progression using low passage primary melanocyte and melanoma cultures and two progression tissue microarrays.

## RESULTS

**Figure 1:**
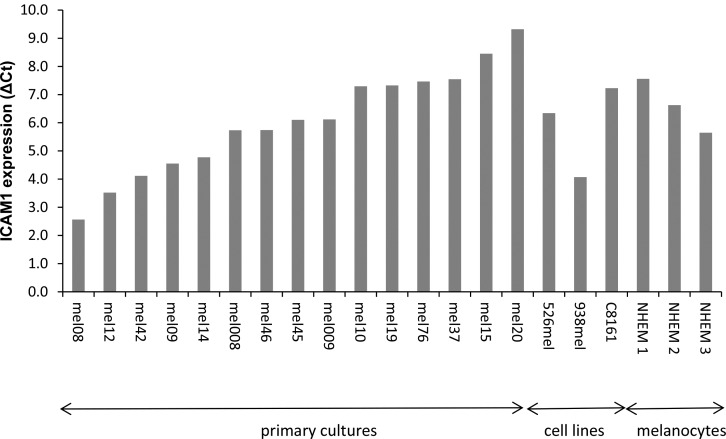
ICAM-1 expression in cultured melanocytes and melanoma cells The expression of ICAM-1 was tested with real-time quantitative PCR in 15 low-passage patient-derived metastatic melanoma cultures, 3 melanoma cell lines and 3 cultures of normal melanocytes. Results are expressed as ΔCt, normalized to GAPDH expression.

To recapitulate previous similar studies, with the exception of focusing mostly on low passage cultures, the expression of ICAM-1 in metastatic melanoma cells was assessed by qRT-PCR using 15 low-passage patient-derived metastatic melanoma cultures, 3 metastatic melanoma cells lines and 3 cultures of normal melanocytes. Similarly to the equivocal available data from the literature, ICAM-1 expression was heterogeneously expressed in all three categories without any clear trend (Figure 1). Notably, mRNA expression analysis cannot distinguish between cytoplasmic and membrane expression.

**Figure 2:**
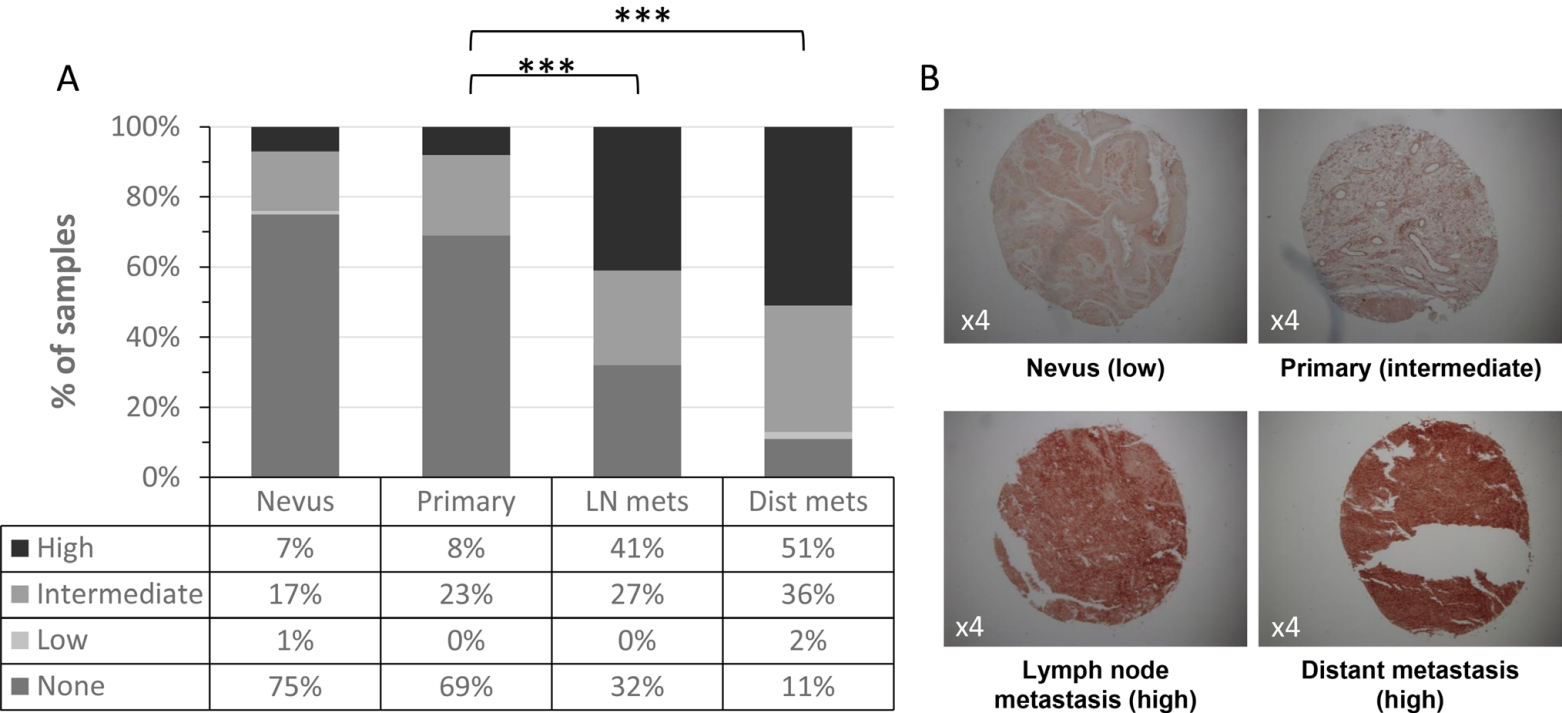
ICAM-1 expression with melanoma development and progression (**A**) ICAM-1 membrane expression was analyzed in melanoma progression TMA comprised of nevi, primary tumors, lymph node (LN) metastases and distant metastases. Intensity staining of ICAM-1 was scored as none, low, intermediate or high; (**B**) Representative staining patterns (×4) of ICAM-1 in melanocytic specimens.

ICAM-1 is an intercellular molecule, which exerts its role by binding to its receptor LFA-1. To take into account cellular location and avoid culture biases, we profiled membrane expression by using the progression TMA which was purchased from the NCI. Due to technical reasons (i.e., folded specimens or tissue loss), only 75 benign nevi, 61 primary tumors, 37 lymph node metastases and 55 metastases from other locations were analyzed for ICAM-1 immuno-staining intensity. ICAM-1 expression profile in this TMA is illustrated in Figure 2A. Clearly, staining intensity increases with disease progression. For example, specimens with high intensity rates comprised only 7% of the nevi, as compared to 51% of the distant metastases. The increase in ICAM-1 expression is statistically significant. While ICAM-1 expression in primary tumors was similar to that of nevi (*p =* 0.44), it was lower than ICAM-1 expression in both lymph node and in distant metastases (*p <* 0.001 for either). ICAM-1 expression in distant metastases demonstrated a trend towards a higher level than in lymph node metastases which was not significant statistically (*p =* 0.09). These data point to the progressive upregulation of ICAM-1 expression, which is particularly evident upon the initial regional spread to the lymph nodes. Representative staining patterns of specimens (×4) are shown in Figure 2B.

We further tested ICAM-1 expression on our in-house designed TMA of paired melanoma specimens from different disease stages, in order to neutralize potential bias of inter-patient variations. Due to uninterpretable spots, staining results of the entire progression spectrum (primary tumor, lymph node metastasis and distant metastasis) were available for only 7 patients of the original 12. Patients with partial spectrum included 3 patients who used to have full progression spectrum and 2 patients who originally had only partial spectrum. Two patients who originally had full spectrum were excluded due to uninterpretable spots for 2 or more samples. Overall, the histopathology slide represented 12 patients with 33 samples out of the original 42 (79%). A 21% sample loss rate is similar to those reported by others (10% to ∼20%) [16]. The clinical and pathological characteristics for these 12 patients are summarized in Table 1.

**Table 1:** Clinical-pathological characteristics of patients comprising the patient-paired TMA

Patient number	Age	Gender	BRAF mutation status	Ulceration of primary tumor	Location of primary tumor	Location of lymph node metastases	Progression from primary to LN metastases (months)	Location of distant metastases	Progression from LN metastases to distant metastases (months)	Overall survival (months)
**1**	66	F	WT	Y	Vulva	Inguinal	0	Small bowel	–9.1	35.8
**2**	70	F	WT	N	Upper back	Axilla	0	Sub-cutaneous	16.5	34
**3**	83	F	WT	UNK	Leg	Thigh	16	Sub-cutaneous	1.2	37.2
**4**	34	M	WT	Y	Leg	Inguinal	1	Sub-cutaneous	40.7	99.5
**5**	59	M	WT	UNK	Conjunctiva	Neck	17.7	Retroperitoneum	14.5	51.1
**6**	60	M	UNK	UNK	Flank	Axilla	13.4	Supraclavicular LN	0	23.7
**7**	71	M	WT	UNK	Shoulder	Axilla	0	Muscle	3.9	11.7
**8**	62	F	UNK	Y	Flank	Axilla	12.2	Sub-cutaneous	–12.17	17.2
**9**	51	F	UNK	Y	Buttocks	Inguinal	0.63	Sub-cutaneous	3.3	16
**10**	43	F	WT	Y	Vagina	Inguinal	5.7	Sub-cutaneous	0	24.2
**11**	85	M	WT	Y	Scalp	Neck	1.9	Salivary gland	10.1	47.2
**12**	50	F	WT	Y	Vulva	Inguinal	99.4	Lung	–59.9	Alive for 151 months

WT stands for Wild Type; UNK stands for Unknown; LN stands for Lymph Nodes.

F-female, M-male.

**Figure 3:**
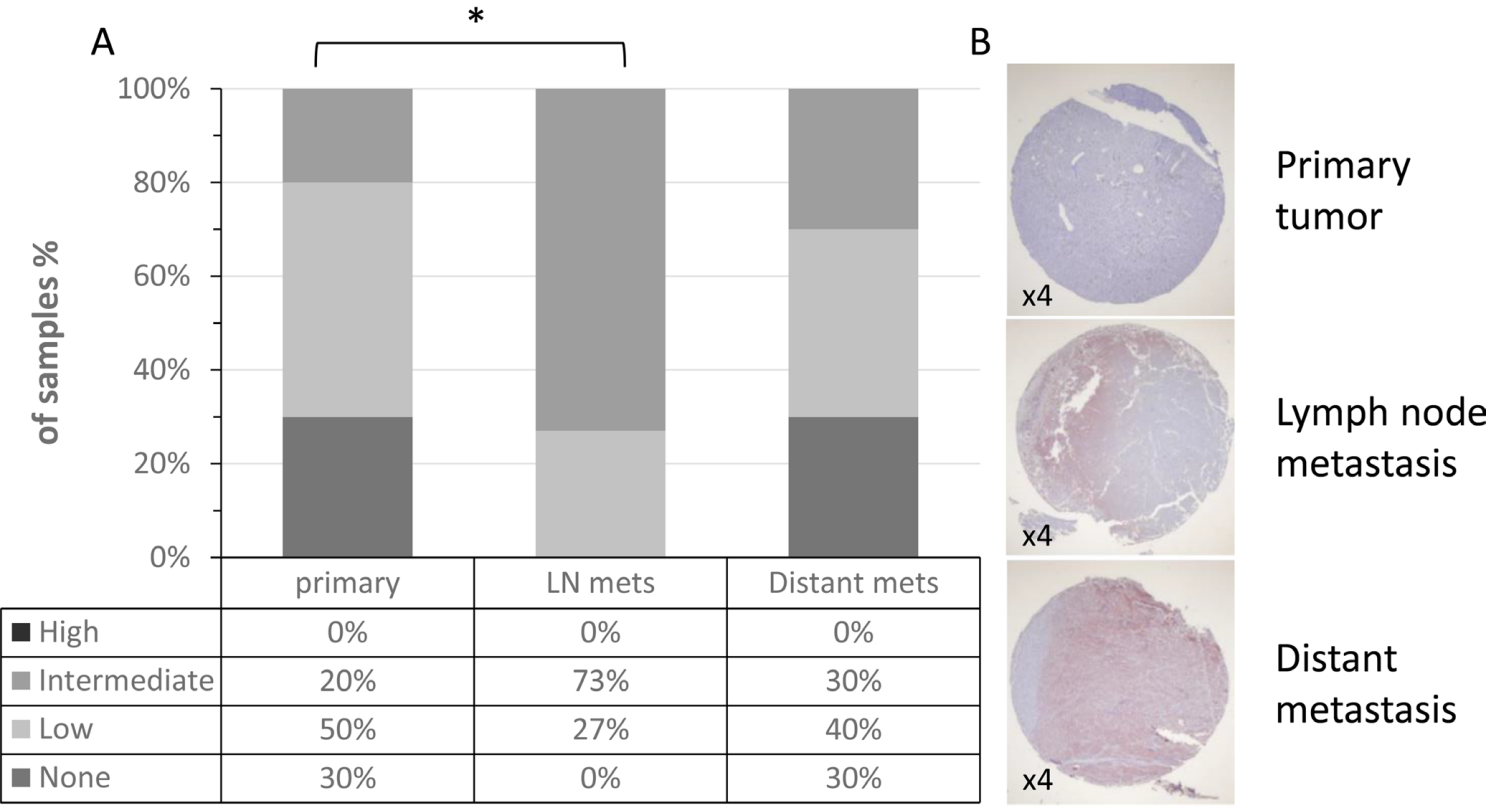
ICAM-1 expression in patient paired samples of disease progression (**A**) ICAM-1 membrane expression was analyzed in our in-house TMA comprised of paired primary tumors, lymph node (LN) metastases and distant metastases. Intensity staining of ICAM-1 was scored as none, low, intermediate or high. (**B**) Representative staining patterns (×4) of ICAM-1 in melanocytic specimens.

As illustrated in Figure 3A, lymph node metastases had a significantly higher ICAM-1 expression than primary tumors (*p =* 0.02). Interestingly, lymph node metastases seem to have higher ICAM-1 expression than distant metastases but this difference was not statistically significant (*p =* 0.068). These results concur with those observed with the TMA purchased from the NCI. A representative staining of all three disease stages from one patient is shown in Figure 3B. There was no significant difference in ICAM-1 intensity between primary tumors of cutaneous or mucosal origin (*p =* 0.8). Additionally, there was no significant difference in ICAM-1 intensity in distant metastases in soft tissue versus visceral organs (*p =* 0.4). Finally, an inverse correlation was suggested between ICAM-1 intensity on primary or distant metastasis and progression to Stage III or to death, respectively (*p =* 0.075 and *p =* 0.08, respectively). ICAM-1 intensity on lymph node metastases was not significantly associated with progression to Stage IV or survival (*p =* 0.13 and *p =* 0.34, respectively).

## DISCUSSION

ICAM-1 expression has been shown to correlate with the metastatic potential of melanoma. Our results show the expression of ICAM-1 at the mRNA level in cultured primary melanoma and cell lines, as well as in melanocytes, is heterogeneous with no significant difference in ICAM-1 expression between benign and malignant lesions (Figure 1). However, at the protein level, a significant increase in ICAM-1 membrane expression along the metastatic progression is observed in two independent TMAs (Figures 2–3). This discrepancy could be explained by the different specimens analyzed (i.e., cultured cells versus paraffin embedded tissues) as well as by the different detection methods applied (i.e., qRT-PCR versus immunohistochemistry). Protein but not mRNA level studies take into account post-transcriptional and post-translational processes, thus the results of the immunohistochemistry are more reliable. Even though the mRNA and IHC studies were not done on the same samples, the substantial amount of cases in the TMA from the NCI and the pairing of samples in our in-house TMA provide statistical confidence for the validity of this observation.

Our findings are in accordance with previous reports showing higher ICAM-1 expression in metastases than in benign and primary lesions [11, 14]. The level of ICAM-1 expression on melanoma cells correlates with tumor thickness and risk of metastases [10–14]. Here we specifically show that ICAM-1 is mostly upregulated in the first site of metastasis, i.e. the lymph node, with no further statistically significant increase in ICAM-1 expression in distant metastases. This suggests that melanoma cells upregulate ICAM-1 in early stages of metastasis to enable at least regional spread. This progressive upregulation is confirmed for the first time in individual paired samples. Indeed, we observed a potential link between the expression intensity of ICAM-1 in primary melanoma and progression to Stage III. There was no association between ICAM-1 in lymph node metastases and progression to Stage IV or overall survival. An additional potential link between ICAM-1 in distant metastasis and lower survival was suggested. It is still unclear why there may be an association between prognosis and ICAM-1 expression on distant metastases but not lymph node metastases. The main study limitation is the small number of included patients. This could highlight the effect of certain subpopulations, for example: a) 9 of 12 samples are BRAF wild type, with the BRAF status of the remaining three unknown; or b) ulcerated primary, which is represented here in 7 of 12 specimens. It should be mentioned that our confirmatory studies were done with different antibodies as compared to earlier studies. In addition, the studies performed on the TMA obtained from the NCI and the patient-matched TMA we generated, were analyzed using anti ICAM-1 antibodies of different lots. This may account for the different intensities reported for the TMAs.

These results may seem to contradict the fact that ICAM-1 strengthens CTL-melanoma cell interactions which would lead to more efficient elimination of the tumor cells and better prognosis [17, 18]. Several possible mechanisms have been suggested to explain this contradiction; ICAM-1 on melanoma cells forms aggregates with leukocytes, allowing their dissociation from the tumor and ultimately their metastatic spread [19, 20]. An alternative explanation is that soluble ICAM-1, shed from melanoma cells, competes with membrane-bound ICAM-1, thus preventing the interaction and destruction of tumor cells by effector cells [21, 22].

## MATERIALS AND METHODS

### Cells

The melanoma lines 526 mel and 938 mel (obtained from Dr. Steven Rosenberg, National Cancer Institute, Bethesda, MD), C8161 (obtained from Dr. Marry Hendrix, Children’s Memorial Research Center, Chicago, IL) and normal human epidermal melanocytes (NHEM; PromoCell, Heidelberg, Germany) were maintained as previously described [8]. The 15 primary cultures derived from surgically removed metastatic melanoma specimens were established and cultured as previously described [23].

### RNA isolation and reverse transcription

Total RNA was isolated from melanoma lines, melanocytes and primary cultures using Tri Reagent (Sigma-Aldrich, Rehovot, Israel), and cDNA was generated by Universal Transcriptor cDNA master (Roche Diagnostics, Basel, Switzerland), according to the manufacturer’s instructions.

### Quantitative real-time PCR (qRT-PCR)

Primers (Sigma-Aldrich) were designed according to Primer-Express software guidelines (Applied Biosystems). The qRT-PCR reactions were run in triplicates LightCycler 480 system (Roche). Gene transcripts were detected using LightCycler 480 SYBR Green I Master (Roche), according to the manufacturer’s instructions. Reactions were normalized to GAPDH endogenous control. Expression was calculated as ΔCt. The detailed sequences of primers used: ICAM-1-F 5′-TGCAGACAGTGACCATCTACAGC; ICAM-1-R 5′-TCACCTCGGTCCCTTCTGAG; GAPDH-F 5′-TGCACCACCAACTGCTTAGC; GAPDH-R 5′-GGCATGGACTGTGGTCATGAG.

### Progression TMA

Progression tissue microarray (TMA) slides were provided by the NCI CDP and included 98 benign nevi, 73 primary tumors, and 41 lymph node metastases and 72 metastases from other locations. Other investigators may have received slides from these same array blocks. Immunohistochemical staining was performed on the TMA samples using a commercially available polyclonal rabbit anti-ICAM-1 antibody (Prestige Antibodies, Sigma Aldrich, Rehovot, Israel) according to standard procedures. A blinded assessment of ICAM-1 expression was conducted by an expert pathologist (IB). For each sample, intensity of ICAM-1 membrane expression was scored as none, low intensity, intermediate or high. Digital images were captured with Olympus BX51 microscope.

### Progression TMA of paired samples

Progression TMA of paired samples from the same patient was designed in-house as a template for the assessment of the melanoma progression process. Formalin-fixed, paraffin-embedded paired tissue samples of primary tumors, lymph node metastases and distant metastases were collected from 12 patients. Paired samples from a patient with a primary tumor and a lymph metastasis, and another patient with lymph node and distant metastases, were further included along with seven normal liver tissue samples and 3 normal muscle tissue samples which were used for orientation and control. Each tissue sample was initially stained with Hematoxylin and Eosin (H&E) and representative areas of tumors were marked by an expert pathologist (IB). Accordingly, three 2mm diameter tissue cylinders were punched out from each tumor block. The cylinders were deposited into a recipient block using Manual Tissue Arrayer MTA-1 (Beecher Instruments Inc., Sun Prairie, WI, USA). Tumor sample triplicates were used as a means of overcoming tumor heterogeneity, as triplicate arrayed-derived data demonstrate a concordance of over 95% with full-section-derived data [24]. Post array construction, a 4 µm section was H&E stained to confirm the histological quality. A consecutive 4 µm section was used for immunohistochemical staining using anti-ICAM-1 antibody, as described above. Each spot was scored by a blinded expert pathologist (IB) according to the same staining intensity scale used in the progression TMA, i.e. none, low intensity, intermediate or high. Uninterpretable cores due to loss of the tissue or excessive background staining were excluded from the analyses.

### Ethics

All studies involving patient-derived material were approved by the Institutional Review Board of Sheba Medical Center.

### Statistical analysis

The Mann Whitney Test was used to analyze the data of TMA purchased from the NCI, while the Wilcoxon Signed Rank Test, was used to analyze the data of the patient-paired TMA. Additionally, for the paired-TMA, the Mann Whitney Test was used to assess the correlation between staining intensity and tumor site while the one-way ANOVA test was used to assess the correlation between staining intensity and time to disease progression. Statistical tests were performed with STATA (STAT Corp, STATA Statistics/Data analysis for Windows, Version 15.0, College Station, Texas, USA). Significance was defined as a *P* value of < 0.05.
